# The combination of perception of other individuals and exogenous manipulation of arousal enhances social facilitation as an aftereffect: re-examination of Zajonc’s drive theory

**DOI:** 10.3389/fpsyg.2015.00601

**Published:** 2015-05-07

**Authors:** Masatoshi Ukezono, Satoshi F. Nakashima, Ryunosuke Sudo, Akira Yamazaki, Yuji Takano

**Affiliations:** ^1^Department of Psychology, Meiji Gakuin University, Minato-ku, Japan; ^2^NTT Communication Science Laboratories, Human Information Science Laboratory, Atsugi-shi, Japan; ^3^Core Research for Evolutional Science and Technology, Japan Science and Technology Agency, Atsugi-shi, Japan; ^4^Graduate School of System Life Sciences, Kyushu University, Fukuoka, Japan; ^5^Department of Liberal Arts, Hiroshima Bunka Gakuen University, Hiroshima, Japan; ^6^Center for Baby Science, Organization for Research Initiatives and Development, Doshisha University, Kyoto, Japan

**Keywords:** social facilitation, arousal, drive theory, social perception, observation

## Abstract

Zajonc’s drive theory postulates that arousal enhanced through the perception of the presence of other individuals plays a crucial role in social facilitation (Zajonc, 1965). Here, we conducted two experiments to examine whether the elevation of arousal through a stepping exercise performed in front of others as an exogenous factor causes social facilitation of a cognitive task in a condition where the presence of others does not elevate the arousal level. In the main experiment, as an “aftereffect of social stimulus,” we manipulated the presence or absence of others and arousal enhancement before participants conducted the primary cognitive task. The results showed that the strongest social facilitation was induced by the combination of the perception of others and arousal enhancement. In a supplementary experiment, we manipulated these factors by adding the presence of another person during the task. The results showed that the effect of the presence of the other during the primary task is enough on its own to produce facilitation of task performance regardless of the arousal enhancement as an aftereffect of social stimulus. Our study therefore extends the framework of Zajonc’s drive theory in that the combination of the perception of others and enhanced arousal as an “aftereffect” was found to induce social facilitation especially when participants did not experience the presence of others while conducting the primary task.

## Introduction

Imagine you are preparing for an exam among customers in a cafe. In such a situation, you may feel that you are working more efficiently than if you were working alone at home. That people tend to perform tasks more efficiently with other individuals present than when performing alone is a phenomenon generally known as “social facilitation” (Allport, 1924; Katz and Schanck, 1938). Social facilitation has been widely reported to occur across species, such as in insects (Chen, 1937; Prokopy and Duan, 1998; Chabaud et al., 2009), birds (Zentall and Hogan, 1976; Ogura and Matsushima, 2011), rodents (Simmel, 1962; Zentall and Levine, 1972), and monkeys (Harlow and Yudin, 1933; Schiml et al., 1996), suggesting that the phenomenon has a common phylogenetically old evolutionary basis.

Allport (1920) was the first to empirically demonstrate the social facilitation phenomenon, which he did by showing that the performance of a word association task improved when a person worked with other individuals relative to performing the task alone. At the same time, he demonstrated the phenomenon known as “social inhibition,” where the performance of a difficult task is attenuated when it is performed with other individuals present relative to the case of its being performed alone (Allport, 1924; Katz and Schanck, 1938). Subsequent studies showed that social facilitation can occur not only when people perform tasks with a “co-actor” who works with them but also when people perform the task with an “observer” who does not work with them (Dashiell, 1930). Researchers have classified social facilitation into two subcategories on the basis of the context of other individuals’ presence: “the co-action effect,” in which task performance is facilitated by concurrent action of other individuals, and “the audience effect,” in which it is facilitated by the presence of an evaluative observer (Bond and Titus, 1983; Harkins, 1987).

### Zajonc’s Drive Theory

The mechanism of social facilitation has been mainly explained in terms of Zajonc’s drive theory (Zajonc, 1965). Drive theory postulates that the arousal level and drive heightened through the perception of the presence of other individuals induces a dominant response of the performer on the task: if the dominant response has already been learned by the performer, it elicits social facilitation, whereas if it has not been experienced, it elicits social inhibition. The results of a number of studies support the main idea of drive theory, and many researchers have considered that arousal enhanced through the perception of the presence of other individuals plays a crucial role in social facilitation (Zajonc, 1980; Bond and Titus, 1983; Guerin, 1993). Heightened arousal based on the perception of others in the social facilitation literature has been examined by using self-reports (McKinney et al., 1983) or several physiological indices such as heart rate (HR; Amoroso and Walters, 1969), palmar sweat (Elliot and Cohen, 1981), and electrodermal (Borden et al., 1976), and cardiovascular responses (Blascovich et al., 1999). According to a meta-analysis (Mullen et al., 1997), when these indices are used, the presence of others in the co-action and audience conditions significantly elicits an arousal level in the performer; however, the mere presence of other individuals does not affect the arousal level measured by self-reports.

Although Zajonc claimed that arousal heightened through the perception of others causes social facilitation, it is unclear whether the perception of the mere presence of other individuals is sufficient to induce arousal enhancement or not. Cottrell et al. (1968) suggested in their evaluation apprehension hypothesis that social facilitation occurs as a result of arousal enhancement through evaluation apprehension—the performer’s perception of being evaluated by others—but that it does not occur with the mere presence of others. In fact, some previous studies have shown that social facilitation occurs as a result of the manipulation of evaluation apprehension (Henchy and Glass, 1968; Good, 1973). On the other hand, other studies have shown that the mere presence of other individuals is sufficient to induce social facilitation (Markus, 1978; Schmitt et al., 1986). Thus, whether the mere presence of others is sufficient to produce social facilitation or not is still being debated.

Both drive theory and the evaluation apprehension hypothesis assume that the elevation of arousal as a result of the perception of others causes social facilitation. After reviewing the social facilitation literature, Aiello and Douthitt (2001) concluded that drive theory, which provided some common concepts that have served as the bases of various subsequent theories, has the highest description rate for the overall research results.

However, the validity of the postulated process of social facilitation in Zajonc’s drive theory needs to be evaluated. Although Zajonc’s theory postulates that the process of social facilitation is that performance is affected by arousal enhancement due to the perception of others, the relationships among these three processes (perception of others, arousal enhancement, and social facilitation) is still unclear. In particular, to our knowledge, no study has directly examined the relationships between arousal enhancement and social facilitation.

In addition, though previous studies have implied that arousal enhancement as a result of the perception of others produces social facilitation, it is unclear whether arousal enhancement due to the perception of others is qualitatively different from that by some other means, such as exercise. To examine what the effect of arousal enhancement on social facilitation is, we need to manipulate arousal directly in some way other than the perception of others. Consequently, by manipulating an exogenous factor other than the presence of others, we examined whether the elevation of arousal causes social facilitation even when the presence of others itself is not sufficient to elevate arousal levels.

### Present Study

To investigate the issues mentioned above, we had participants perform a simple addition task twice: First as a baseline measurement and then as post-manipulation measurement. We chose the addition task because it is quite easy to conduct and well-learned in general and therefore suitable for producing social facilitation rather than social inhibition (Bond and Titus, 1983). In the baseline measurement, all participants performed the addition task alone without any manipulations. After performing the task, they measured the baseline of physiological indices of arousal. They were asked to record the psychological measure of their arousal soon afterward. They subsequently experienced any of one of five conditions: control, observed, greeting, exercise, and observed-with-exercise. For the observed and greeting conditions, we manipulated the presence of the observer. The only difference between them was that the observer gave participants a short greeting in the latter. We employed these different conditions to assess whether the presence or absence of interaction between observers and participants affects the amount of social facilitation. For the exercise condition, we manipulated only the arousal enhancement exogenously with a stepping exercise. For the observed-with-exercise condition, we manipulated both observer presence and arousal enhancement concurrently. For the control condition, we did not manipulate any factors at all. After they had experienced one of these five conditions, participants were again asked to measure their physiological and psychological indices of arousal after manipulation. Then, they performed the addition task as a post-manipulation measurement.

The stepping exercise is well known as an effective method for increasing arousal exogenously (Sanbonmatsu and Kardes, 1988). However, because of the practical problem of simultaneously conducting the stepping exercise and a cognitive task for measuring the amount of social facilitation, we had participants conduct the cognitive task alone in all experimental conditions and manipulated the presence-of-others and exercise factors before they had conducted it. In the main experiment, we therefore examined social facilitation as an aftereffect of social stimulus that resulted from the perception of others.

We predicted that the performance of the addition task in the observed-with-exercise condition would be higher than in any of the other conditions, because, if the cause of arousal enhancement does not matter for social facilitation, the combination of the presence of other individuals and arousal enhancement by the stepping exercise should produce a sizable improvement in performance of that task compared to only the presence of others.

## Main Experiment

### Methods

#### Experimental Design

The experiment was conducted in a one-factor design with the five conditions (control, observed, greeting, exercise, observed-with-exercise) as a between-subject design.

#### Participants

Participants were 110 healthy Japanese undergraduates, graduate students, and alumni and alumnae who had graduated within the last 3 years (42 men and 68 women, age: *M* = 22.05, SD = 1.8). Participants were assigned to one of the five different conditions: control (8 men, 14 women), observed (9 men, 13 women), greeting (8 men, 14 women), exercise (8 men, 14 women), and observed-with-exercise (9 men, 13 women). This experiment was conducted in accordance with the ethical code of the Japanese Psychological Association and the research protocol of the experiment was approved by the Ethical Practices Committee of Meiji Gakuin University. Written informed consent was obtained from all participants before the experiment.

#### Procedure

**FIGURE 1:**
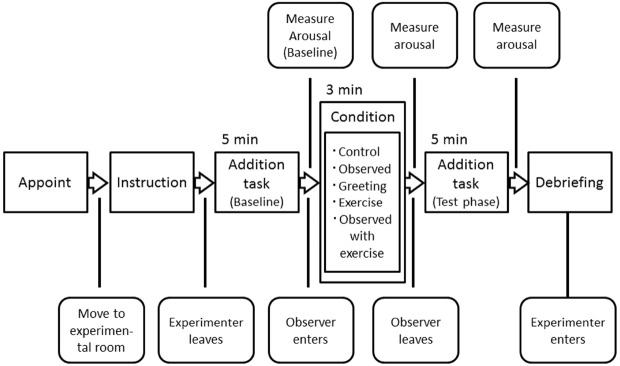
**Flowchart of the experimental procedure.** The experimenter met a participant outside the experimental room and then they entered it together. The experimenter did not talk with the participant during this time. The participant was asked to conduct the task alone in accordance with a script that described the experimental procedure. And the experimenter demonstrated how to use the automatic sphygmomanometer and do the single-digit addition task. After providing participants with instruction, the experimenter left the experimental room. The participant conducted the addition task for the baseline. After the baseline session, the participants measured their arousal level using the automatic sphygmomanometer and by self-reports. Then, they spent 3 min in any one of the five conditions (see Methods), which were assigned at random by the experimenter. Before the test session, the participants measured their arousal levels. Then, they calculated the addition task as a test. Finally, participants measured their arousal levels again.

Participants were tested individually. In order to eliminate the effect of experimenter presence, they conducted the task alone following a script that described the experimental procedure, and their behavior in the experiment was recorded with a video camera (30 fps, Handycam HDR-CX560, Sony Corp.) to check whether they had conducted the task appropriately. We informed them that their behavior would be recorded with a video camera solely for the purpose of checking whether they were conducting the experiment appropriately, because previous studies have shown that such instruction prevents the effect of the presence or absence of the video camera itself on task performance (Aiello and Svec, 1993). In addition, they were told that an experimenter may enter the room to check whether there were any problems during experiment, and they were also instructed to conduct the experiment based on the script and not to be concerned about the experimenter’s entering or leaving the room. The general flow of our experiment is shown in Figure 1.

We used a simple addition task based on the Uchida-Kraepelin test (Kuraishi et al., 1957), which is a questionnaire modified from the Kraepelin arithmetic test (Kraepelin, 1902). The task was to add one number to the next number and write the answer in the margin between each number. We asked participants to answer with only single digits (for example, for 7 + 4, the answer is 1). Single-digit numbers for questions were printed in a 19 × 30 matrix on a sheet of paper. We calculated the index of task performance facilitation in the addition task by subtracting the total number of calculated numerical values in the baseline phase from that in the test phase for each participant in each condition.

As physiological indices of the arousal level, blood pressure and HR were measured with an automatic sphygmomanometer (UB-328A, A&D), which can measure systolic blood pressure (SBP), diastolic blood pressure (DBP), and HR. Participants used this apparatus as follows. First, they sat at a table and wrapped the band of the apparatus around their wrist at the measuring point. They then stabilized their arm by putting the elbow on the table and held their hand above the heart. Finally, they pushed the start button and stayed at rest, and after a period of time, the SBP, DBP, and HR values were displayed on the screen of the apparatus. The apparatus is a home electric healthcare appliance and therefore easy to handle alone. Participants were asked to enter the SBP, DBP, and HR on log sheets and to repeat the measurement if an error message was displayed on the screen. Before the experiment, an experimenter demonstrated how to use the automated sphygmomanometer and do the single-digit addition task. Participants could do the experiment at their own pace after the experimenter had left the experimental room.

At the beginning of the experiment, they were asked to do the addition task for 5 min as a baseline session. Then they measured their SBP, DBP, and HR as the baseline of their arousal level and entered the values on their log sheets. They also used a five-point scale [excited (5) ∼ calm (1)] as a self-report of their arousal level. After that, they experienced for 3 min any one of the five conditions (control, observed, greeting, exercise, observed-with-exercise), which were assigned at random by the experimenter. After experiencing each condition, participants were again asked to measure their SBP, DBP, and HR and use the five-point scale as indices of their arousal level after manipulation. Then, they did the addition task as a test session for 5 min. Finally, participants were asked to measure each physiological index and rate their arousal level on the five-point scale once again.

Each condition was as follows. In the control condition, participants were only asked to wait alone without doing anything for 3 min. In the observed condition, a confederate as a stranger entered the experimental room and stayed there for 3 min. In the greeting condition, the manipulation was the same as in the observed condition except that the stranger greeted participants upon entering the experimental room with “Hello!!” and exiting it with “Good luck!” In the exercise condition, participants were asked to do the stepping exercise with a stepstool for 3 min. The pace of the stepping was regulated as fifty times a minute with an electronic metronome so that participants performed 150 steps in total. In the observed-with-exercise condition, a stranger entered the room while participants were doing the stepping exercise and withdrew from the room after 3 min.

We assigned one of 10 confederates (5 men; 5 women) to a participant of the same sex, ensuring that this was their first encounter with each other. Each confederate as a stranger was not given any information about the purpose of the experiment and was only directed to take note of the behavior of participants while sitting in a chair without speaking. The chair was located beside the door on the other side of the room from where participants conducted the addition task. The chair was located approximately 2.5 m from the participant and approximately 1.8 m from the platform for the stepping exercise. In both the observed condition and observed-with-exercise condition, participants only noticed that somebody came into the room from the sound of the door of the room opening and closing. Actually, nobody realized that this was their first encounter with a stranger in the observed condition and observed-with-exercise condition. On the other hand, in the greeting condition, all participants looked back at the strangers because of the greeting and thus realized that this was their first encounter with them.

### Results and Discussion

**FIGURE 2:**
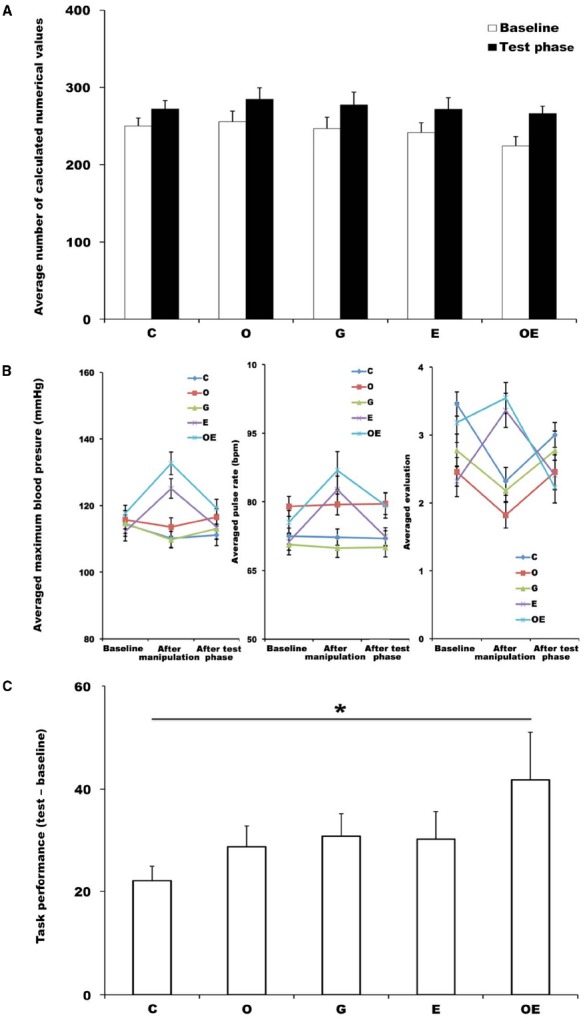
**(A)** Average number of values participants calculated during the addition task in the baseline phase and test phase. Error bars indicate the standard error of the mean in each condition. C, control condition; O, observed condition; G, greeting condition; E, exercise condition; OE, observed-with-exercise condition. *p < 0.05. **(B)** Mean values of SBP, HR, and self-reports at the baseline, after manipulation, and after test phase. Error bars indicate the standard error of the mean in each condition. **(C)** Averaged differences in single-digit addition task performance between the baseline results and the test session in each condition. Error bars indicate the standard error of the mean in each condition. C, control condition; O, observed condition; G, greeting condition; E, exercise condition; OE, observed-with-exercise condition. *p < 0.05.

Figure 2A shows the values for the addition task performance during the baseline and test phase. Figure 2B shows the values of physiological and psychological indices during the baseline, after the manipulation, and after the test phase.

#### Manipulation Assessment

To assess the manipulation’s effectiveness for arousal enhancement, we calculated the amounts of change in SBP, HR, and self-reports by subtracting the values after the manipulation from those obtained in the baseline phase. The amounts of change in SBP, HR, and self-reports in each condition are shown in Table 1. A one-way ANOVA was conducted on the values of SBP, HR, and self-reports of the arousal level for each condition.

**TABLE 1:** **The amount of change in arousal levels**.

	**SBP (mmHg)**	**Heart rate (bpm)**	**Self-report**
Control	–4.23 (–1.59)	–0.23 (1.64)	–1.14 (0.22)
Observed	–2.18 (1.94)	0.41 (1.11)	–0.64 (0.15)
Greeting	–4.91 (3.03)	–0.73 (0.81)	–0.59 (0.19)
Exercise	12.91** (1.59)	11.36** (2.28)	1.05** (0.25)
Observed with exercise	15.00** (2.51)	11.32** (2.64)	0.36** (0.31)

Averaged differences in SBP, HR, and self-reports between the baseline results and after manipulation in each condition. The numbers in brackets indicate the standard error of the mean in each condition. Post hoc t-test with Tukey’s correction revealed a significant difference between observed-with-exercise condition and all other conditions except the exercise condition, and between the exercise condition and all other conditions except the observed-with-exercise condition. **p < 0.01.

The results showed that the amounts of change in SBP, HR, and self-reports were significantly different between conditions: SBP, *F*(4,105) = 18.84, *p* < 0.01, ${\text{η}}_{\text{p}}^{\text{2}}$ = 0.42; HR, *F*(4,105) = 11.36, *p* < 0.001, ${\text{η}}_{\text{p}}^{\text{2}}$ = 0.30; self-reports, *F*(4,105) = 14.04, *p* < 0.001, ${\text{η}}_{\text{p}}^{\text{2}}$ = 0.35. A *post hoc* Tukey’s comparison revealed a significant difference between the observed-with-exercise condition and all other conditions (SBP, all *p*’s < 0.001; HR, all *p*’s < 0.001; self-reports, all *p*’s < 0.05) except the exercise condition [SBP, *p* > 0.10, not significant (n.s.); HR, *p* > 0.10, n.s.; self-reports, *p* > 0.10, n.s.]. In addition, it revealed a significant difference between the exercise condition and all other conditions (SBP, all *p*’s < 0.001; HR, all *p*’s < 0.001; self-reports, all *p*’s < 0.001) except the observed-with-exercise condition (SBP, *p* > 0.10, n.s.; HR, *p* > 0.10, n.s.; self-reports, *p* > 0.10, n.s.). The differences among the control condition, observed condition, and greeting condition were also not significant (SBP, *p* > 0.10, n.s.; HR, *p* > 0.10, n.s.; self-reports, *p* > 0.10, n.s.). These results mean that arousal enhancement was different between conditions and that the conditions with exercise heightened the arousal level more than the other conditions.

To assess the appropriate index of arousal, we checked the correlations between SBP, HR, and self-reports. There were significant correlations between all indices (SBP and HR: *r* = 0.47, *p* < 0.001; SBP and self-reports: *r* = 0.53, *p* < 0.001; HR and self-reports: *r* = 0.42, *p* < 0.001). These results mean that the indices of arousal in this study were appropriate for assessing the arousal enhancement.

To validate the homogeneity of the baseline performance of the addition task among conditions, we conducted a one-way ANOVA on the values of the addition task performance in the baseline. The results showed that there were not significant differences among conditions: [*F*(4,105) = 2.46, *p* = 0.50, ${\text{η}}_{\text{p}}^{\text{2}}$ = 0.03, n.s.]. The result means that we could allocate participants to each condition equally in regard to their baseline performance of the addition task. Consequently, the following outcomes showing differences among conditions are attributed to the effect of manipulation but not to the differences in the participant’s baseline performance of the addition task among conditions.

#### Index of Facilitation of Task Performance: Increment of Number of Calculated Values

We calculated an index of the facilitation of task performance in the addition task by subtracting the total number of calculated numerical values in the baseline phase from that in the test phase in each condition. The indices of the facilitation of performance in the addition task for each condition are shown in Figure 2C. A one-way ANOVA was conducted on the values of the facilitation indices for each condition. The results showed that the amount of facilitation in task performance was significantly different between conditions: *F*(4,105) = 2.77, *p* = 0.03, ${\text{η}}_{\text{p}}^{\text{2}}$ = 0.09. A *post hoc* Tukey’s comparison revealed a significant difference between the control condition and the observed-with-exercise condition (*p* = 0.01, *d* = 0.83). In contrast, there were no significant differences between the control condition and any other condition (observed condition, *p* = 0.80, *d* = 0.55, n.s.; greeting condition, *p* = 0.60, *d* = 0.68, n.s.; exercise condition, *p* = 0.66, *d* = 0.55, n.s.). In addition, there were no significant differences between the observed-with-exercise condition and any other condition (observed, *p* = 0.21, *d* = 0.52, n.s.; greeting, *p* = 0.37, *d* = 0.44, n.s.; exercise, *p* = 0.32, *d* = 0.44, n.s.). The differences among the observed condition, greeting condition, and exercise condition were also not significant (all *p*’s > 0.10, n.s.). These results indicate that the task performance in the observed-with-exercise condition was facilitated more than that in control condition. In other words, the combination of the perception of the presence of others and arousal enhancement was required for producing sufficient social facilitation in this study.

#### Effect of Individual Differences in Ability to Perform Addition Task on Social Facilitation

**FIGURE 3:**
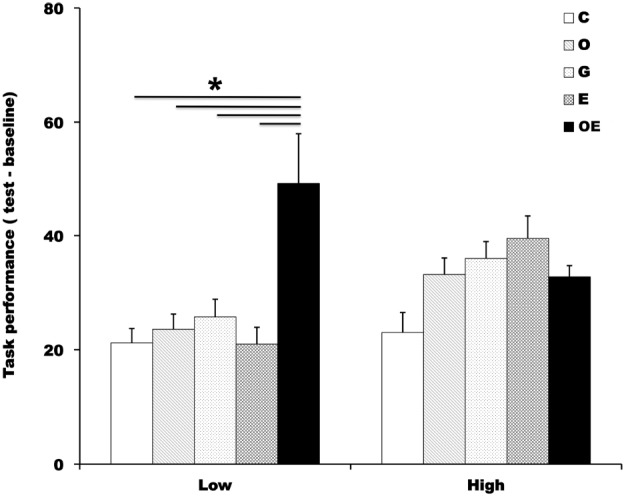
**Participants were divided into a high-score group and a low-score group on the basis of the median value in the addition task in baseline.** The graph shows the average number of values participants calculated during the addition task in the baseline phase and test phase. Error bars indicate the standard error of the mean in each condition. C, control condition; O, observed condition; G, greeting condition; E, exercise condition; OE, observed-with-exercise condition.*p < 0.05.

To examine whether individual differences in ability to perform the addition task affected the amount of social facilitation, we divided participants into the a high-score (*N* = 55) group and a low-score (*N* = 55) group on the basis of the median value of the performance score for the addition task (median = 243, maximum = 413, minimum = 95) in the baseline measurement and calculated an index of the facilitation of task performance for each group (Figure 3).

We conducted a 2 (performance in addition task: high, low) × 5 (condition: control, observed, greeting, exercise, and observed-with-exercise) two-way ANOVA on the values of the facilitation indices. There was a significant main effect of condition: *F*(4,100) = 2.73, *p* = 0.03, ${\text{η}}_{\text{p}}^{\text{2}}$ = 0.1. However, this effect was qualified by performance in the addition task × condition interaction: *F*(4,100) = 2.59, *p* < 0.05, *p* = 0.04, ${\text{η}}_{\text{p}}^{\text{2}}$ = 0.1. A subsequent analysis showed that the simple main effect of condition was significant only in the group with low performance scores: *F*(4,100) = 4.48, *p* < 0.01, ${\text{η}}_{\text{p}}^{\text{2}}$ = 0.15. A *post hoc* Tukey’s comparison showed that a significant difference between the observed-with-exercise condition and all other conditions (control, *p* = 0.007, *d* = 0.95; observed, *p* = 0.03, *d* = 0.85; greeting, *p* = 0.04, *d* = 0.76; exercise, *p* = 0.007, *d* = 0.92). The results indicate that the individual differences in ability to perform the addition task could have been a crucial factor in the amount of social facilitation: Participants who performed poorly in the addition task could have been influenced by the effect of the combination of the perception of the presence of others and arousal enhancement compared to those who performed the addition task well.

#### Increment of Errors in the Task

We calculated the increase in addition task errors as an index of the inhibition of task performance by subtracting the number of errors in the baseline phase from those in the test phase in each condition. A one-way ANOVA was conducted on the values of the indices for each condition. The results showed that the index was not significantly different between conditions: [*F*(4,105) = 2.09, *p* = 0.09, ${\text{η}}_{\text{p}}^{\text{2}}$ = 0.07, n.s.]. The results indicate that the facilitation of task performance described above was not derived from the speed and accuracy trade-off.

In accordance with our prediction, the results showed that the task performance in the observed-with-exercise condition was higher than that in any other condition, particularly for the participants whose skill in the addition task was low in the baseline measurement. This indicates that the combination of the perception of the presence of others and arousal enhancement might be crucial for social facilitation. It also indicates that the combination was sufficient to produce social facilitation even when the participants performed the addition task itself alone after they had experienced the perception of the presence of others and exercise.

However, there is the possibility that the “social facilitation” that we showed in the present study might be different from that in previous studies, because the experimental situation was quite different from that in previous studies. In previous studies, participants conducted the primary task with the presence of others, whereas, in the current study, they conducted the task itself alone after they had experienced the perception of the presence of others. In this regard, contrary to previous studies, the results showed that the perception of the presence of others alone was not enough to facilitate task performance in this study.

One possible interpretation of this result is that the effect of the presence of others was weakened because of the absence of an observer during the primary task. In this case, we would find the effect of the presence of others if an observer were present during the task. Another possibility is that the manipulation of the presence of others in the current study itself was problematic; that is, the presence of others may not have affected task performance. In this case, we would not find any effect of the presence of others even if an observer were present during the task. To examine these possibilities, we conducted an additional experiment with an observer present during the addition task.

We need to mention that another question arises with this experimental setting: does the effect of the combination of the perception of others and arousal enhancement we examined as an “aftereffect” still remain even if an observer is present during the primary task? To answer this question, we compared the increment of the performance in the observed-with-exercise condition to that in the exercise condition, with an observer present during the primary addition task. If the effect of the combination of the perception of others and arousal enhancement still remains, the observed-with-exercise condition might facilitate task performance relative to the observed condition even if an observer is present also during the following addition task itself.

## Supplementary Experiment

In this experiment, we had two main purposes. One was to examine whether the presence of an observer during the addition task facilitates task performance. The other was to examined whether the effect of the combination of the presence of others and arousal enhancement is stable even if an observer is present during the addition task. For these purposes, we set two conditions: observed + observed at test (O + O) and observed-with-exercise + observed at test (OE + O). The manipulation methods for these conditions were the same as in main experiment, except that the conditions now included an observer during the test phase.

### Methods

Forty-four healthy Japanese participated in this experiment (17 men and 27 women, age: *M* = 21.36, SD = 4.46). The task, apparatus, and procedure were the same as in the observed condition and the observed-with-exercise condition in main experiment, except that we manipulated the presence of observers during the test phase. In the supplementary experiment, the observers as confederates were three men who were all strangers to the participants. Participants were randomly assigned to one of the two conditions: O + O (8 men, 14 women) and OE + O (9 men, 13 women).

### Results and Discussion

**FIGURE 4:**
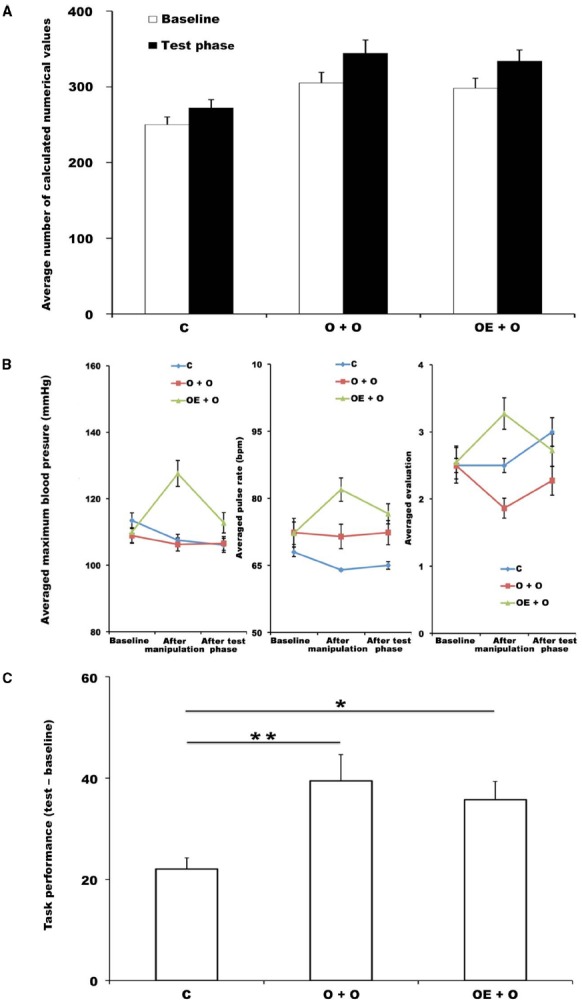
**(A)** Average number of values participants calculated during the addition task in the baseline phase and test phase. Error bars indicate the standard error of the mean in each condition. C, control condition (main experiment); O + O, observed + observed at test condition (supplementary experiment); OE + O, observed-with-exercise + observed at test condition (supplementary experiment). *p < 0.05. **(B)** Mean values of SBP, HR and self-reports at the baseline, after manipulation, and after test phase. Error bars indicate the standard error of the mean in each condition. **(C)** Averaged differences in single-digit addition task performance between the baseline results and the test session in each condition. Error bars indicate the standard error of the mean in each condition. C, control condition (main experiment); O + O, observed + observed at test condition (supplementary experiment); OE + O, observed-with-exercise + observed at test condition (supplementary experiment).*p < 0.05; ** *p* < 0.01.

To assess the amount of arousal enhancement and the increment of task performance, we used the data from the control condition in the main experiment and compared the values for the two conditions in the supplementary experiment (O + O and OE + O) with that for the control condition. Figure 4A shows the values for the addition task performance during the baseline and test phase. Figure 4B shows the values of the physiological and psychological indices during the baseline, after manipulation, and after the test phase.

First, to affirm whether the manipulation of arousal enhancement was appropriate, we calculated the amounts of change in SBP, HR, and self-reports as we did in the main experiment. A one-way ANOVA was conducted on the values of SBP, HR, and self-reports of the arousal level for each condition. The results showed that the amounts of change in SBP, HR, and self-reports were significantly different between conditions: [SBP, *F*(2,63) = 52.78, *p* < 0.01, ${\text{η}}_{\text{p}}^{\text{2}}$ = 0.63; HR, *F*(2,63) = 19.21, *p* < 0.001, ${\text{η}}_{\text{p}}^{\text{2}}$ = 0.38; self-reports, *F*(2,63) = 23.01, *p* < 0.001, ${\text{η}}_{\text{p}}^{\text{2}}$ = 0.42]. A *post hoc* Tukey’s comparison revealed a significant difference between the OE + O condition and other two conditions (SBP, all *p*’s < 0.001; HR, all *p*’s < 0.001; self-reports, all *p*’s < 0.01). The differences between the control condition and O + O condition were not significant (SBP, *p* > 0.10, n.s.; HR, *p* > 0.10, n.s.; self-reports, *p* > 0.10, n.s.).

We calculated an index of the facilitation of task performance in the addition task as we did in the main experiment (Figure 4C). A one-way ANOVA was conducted on the values of the facilitation indices for each condition. The results showed that the amount of facilitation in task performance was significantly different between conditions [*F*(2,63) = 5.32, *p* = 0.007, ${\text{η}}_{\text{p}}^{\text{2}}$ = 0.15]. A *post hoc* Tukey’s comparison revealed a significant difference between the control condition and O + O condition (*p* = 0.008, *d* = 0.93) and between the control condition and OE + O condition (*p* = 0.046, *d* = 0.97). In contrast, there was no significant difference between the O + O condition and OE + O condition (*p* = 0.78, n.s.).

The results indicated that the increment of task performance in the O + O condition and OE + O condition was higher than that in control condition. This supports our prediction that just the effect of the “presence of others” would have sufficient power to facilitate task performance if an observer were present during the addition task.

In contrast, we did not find a significant difference between the O + O condition and OE + O condition. One possible reason is that the effect of the presence of an observer during the primary task was too strong to elevate arousal and therefore masked the effect of the arousal enhancement as an aftereffect.

Another possible reason is the simple ceiling effect on the increment of task performance. To examine whether the performance of the addition task in these two conditions reached the ceiling of task performance, we compared these conditions with the observed-with-exercise condition in the main experiment. A one-way ANOVA was conducted on the values of the facilitation indices for the observed-with-exercise (main experiment), O + O (supplementary experiment), and OE + O (supplementary experiment) conditions. The result showed that there were no significant differences between each condition [*F*(2,63) = 0.31, *p* < 0.10, ${\text{η}}_{\text{p}}^{\text{2}}$ = 0.01, n.s.]. This means that the effect of the presence of an observer during the primary task did not promote task performance relative to that of the combination of the presence of others and arousal enhancement as an aftereffect. In other words, the aftereffect itself was already strong enough to facilitate task performance, and there might be no room for the effect of the presence of an observer during conducting task to improve task performance.

## General Discussion

In the present study, we examined whether the combination of arousal enhancement through a stepping exercise and the perception of the presence of others facilitates the performance of an addition task, even when the perception of others itself does not produce arousal enhancement. The results of the main experiment indicated that the combination of arousal enhancement and perception of others induced the strongest facilitation as an aftereffect, especially in people with low skill in the addition task in the baseline phase. In supplementary experiment, we examined whether the presence of an observer during the addition task facilitates task performance and whether the effect of the combination of the presence of others and arousal enhancement is stable even when an observer is present during the addition task. The results showed that the presence of others during the task is enough on its own to produce facilitation of task performance, regardless of the manipulation of their presence and arousal enhancement as an aftereffect.

### Manipulation Assessment in Arousal Level

The results indicate that the manipulation of arousal enhancement in the main and supplementary experiments was appropriate. First, the exercise condition and observed-with-exercise condition were significantly different from any other condition, whereas the observed condition and greeting condition were not significantly different from the control condition in the main experiment. In addition, the OE + O condition in the supplementary experiment was significantly different from the control condition, whereas the O + O condition was not. This means that the arousal level was higher in both the exercise and observed-with-exercise conditions than in the other conditions. Second, we conducted an additional analysis of the SBP and HR and found that both of them were higher after the manipulation than in the baseline phase and at the end of the experiment only for those two conditions. This means that arousal levels in those conditions were higher after manipulation than those at the baseline.

### Expanding Zajonc’s Drive Theory

The results partially support our hypothesis that the performance of the addition task in the observed-with-exercise condition would be higher than in any other condition. The results of the main experiment showed that only the observed-with-exercise condition was significantly different from the control condition in task performance, whereas the differences in task performance between other conditions and the control condition were not significant. These findings indicate that the perception of the presence of others was not sufficient to elevate the arousal level compared with the control condition or to facilitate the performance of the task even when a stranger greeted the participant. In addition, they reveal that exogenous manipulation of the arousal level by the stepping exercise was insufficient for facilitating task performance, though it was sufficient for enhancing the arousal level.

On the other hand, the combination of the presence of another individual and the stepping exercise increased both task performance and the arousal level significantly compared with the control condition. In other words, the combination of the presence of others and the elevation of arousal induced the strongest social facilitation effect even when the method of arousal enhancement was unrelated to the perception of others and the method itself had a small impact on the performance of the task. These results support the hypothesis in Zajonc’s drive theory that both perception of others and enhancement of arousal are necessary in order to induce social facilitation (Zajonc, 1965, 1980). However, at the same time, they indicate that the arousal enhanced through the perception of others might not be necessary for social facilitation. Even so, participants in the O + O condition in the supplementary experiment did not show enhanced arousal. Our study therefore extends the framework of Zajonc’s drive theory in that the combination of perception of others and arousal enhanced through an extrinsic factor was found to induce social facilitation even when the perception of others itself does not elevate the arousal level.

We have to mention that we did not find significant differences between the observed-with-exercise condition and observed or greeting condition. We therefore cannot confidently conclude from only the results of this study that the combination of the perception of the presence of others and elevation of arousal produces the strongest social facilitation. However, when we divided participants into high and low groups based on their ability in the task in the baseline phase, we found significant differences between the observed-with-exercise condition and all other conditions—which included the observed and greeting conditions—in the group of participants who had poor skill in the addition task. This means that, at least for participants who did not perform the task efficiently in the baseline phase, the combination of the perception of the presence of others and arousal enhancement was required for producing social facilitation.

In the meantime, for the group of participants who had good skill in the addition task, we did not find significant differences between the observed-with-exercise and the other conditions. This might simply be because there was little range in facilitating task performance for these participants: they had already conducted the task efficiently in the baseline phase. In other words, the effect of the combination of the presence of others and arousal enhancement might have appeared even in this group if their task performance in baseline had not reached a ceiling. Examining this possibility with other kinds of tasks requires further study.

It is worth pointing out that the number of calculated numerical values is not attributable to the trade-off between the speed and accuracy of the calculation. There were no significant differences between any pair of conditions in terms of the number of errors in the addition task, though there were significant differences among the observed-with-exercise condition and all other conditions in terms of the number of calculated values. This means that the number of calculated values genuinely represents the occurrence of social facilitation, as was described earlier. In addition, the number of errors in the task mean that we did not see the social inhibition that some previous studies have shown (Bond and Titus, 1983). This may because the task was too simple for the participants and well learned.

### Social Facilitation as “Aftereffect of Social Stimulus”

In addition, it is important that social facilitation occurred without the presence of others during the cognitive task: in our study, social facilitation was an aftereffect as a result of the perception of others immediately before the task. Previous studies have examined the effect of the presence of others on social facilitation concurrently with having participants conducta task (Guerin, 1993). In our study, we had no difficulty manipulating the presence-of-others factor concurrently with the single-digit addition task in the same manner as in previous studies; however, we had difficulty manipulating the stepping exercise concurrently with single-digit addition task. We therefore manipulated the presence of others and the stepping exercise before the participants had conducted the single-digit addition task to control the effect of extraneous variables on task performance between each condition. Namely, the results indicate that the effect of the presence of others and of the enhancement of arousal on social facilitation could last longer than expected from the results of conventional research.

However, a remaining issue is whether the mechanism of “social facilitation” as an aftereffect of social stimulus that we suggested is actually the same as that in previous studies. In fact, we were concerned about whether our manipulation method for the presence of observers was appropriate because we did not find a significant difference between the observed condition and control condition in the main experiment. To untangle the concern, we conducted the supplementary experiment, in which we added the presence of an observer during the primary addition task. In the results, we found that the increment of the performance in both the O + O and OE + O conditions was higher than that in the control condition. This means that the presence of an observer during the task was enough on its own to produce facilitation of task performance. Therefore, it is considered that our manipulation of the presence of others was appropriate. In addition, we did not find any significant differences in the increment of task performance between the observed-with-exercise condition in main experiment and the OE + O condition in the supplementary experiment. This means that the effect of combination of presence of other and arousal enhancement as an aftereffect was at least as strong as the effect of the presence of an observer during the primary task, though there was also the possibility that the effect of the presence of others during the task caused the simple ceiling effect on the increment of task performance. Although the results may not arise from the same mechanisms, the mechanisms are at least similar in the point that they produce facilitation of task performance. We cannot, however, directly resolve the issue from the results of this study alone. Further research will be needed to examine the mechanism of social facilitation as an aftereffect of social stimulus.

### Limitation of the Study

An issue in this study is sample size. As we described earlier, in the main experiment, social facilitation did not occur as a result of only the perception of the presence of others or only arousal enhancement. We found, however, that the effect sizes between the control condition and the observed, greeting, or exercise condition were moderate (observed, *d* = 0.55; greeting, *d* = 0.68; exercise, *d* = 0.55). This means that a sufficient increment of sample size might show facilitation of performance with only perception of the presence of others or only arousal enhancement.

We should mention that we examined the relationships between the perception of others and arousal, not those between the perception of others and the postulated core concepts in previous studies, such as motivation and drive. Some studies have suggested that an increase in motivation of participants in addition to that in arousal or drive elicits social facilitation or social inhibition. For example, Carver and Scheier (1981) demonstrated that the motivation for task performance due to self-evaluation for appraisal from other individuals produces social facilitation or inhibition. In addition, Blascovich et al. (1999) showed that the motivational state of participants affects arousal levels, such as cardiac response, to produce social facilitation. The purpose of our study was to examine the relationships between arousal and social facilitation; therefore, we cannot refer to the relationships between perception of others, arousal, drive, and motivation. Further studies are needed in order to clarify the relationships among the core concepts related to social facilitation.

Finally, we should note that our sample includes Japanese participants only. Although Zajonc’s drive theory itself does not suppose cultural differences in social facilitation, there is the possibility that the cultural background of participants affects the amount of social facilitation. A great deal of research in the field of cultural psychology have shown that the features of cognition and social behavior differ between the people in the east and west (for reviews, see Markus and Kitayama, 1991; Nisbett et al., 2001). For example, one study showed that the effect of social cues, such as gaze, on choice behavior was different between Japanese and Americans (Kitayama et al., 2004). In addition, Karau and Williams (1993) have indicated from their meta-analysis that the amount of social loafing is more salient in westerners than easterners. In the future, more research will need to focus on such cultural differences in social facilitation.

## Conclusion

In summary, the present study suggests that social facilitation could be partly explained by Zajonc’s theory. However, it was unclear whether an increase of the arousal level due to only the perception of others produces social facilitation. One possibility is that social facilitation is generated by the mere combination of the increase of the arousal level through non-social factors and the perception of others, which itself does not increase the arousal level. A misattribution of a causal association between the increase of arousal level and social perception could be a potential mechanism for inducing social facilitation. Further examination of “what arousal is” and “what social perception is” is needed.

## Author Contributions

MU, SN, AY, YT designed the experiment. MU and RS performed the research. MU analyzed the data. MU, SN, and YT wrote the paper.

### Conflict of Interest Statement

The authors declare that the research was conducted in the absence of any commercial or financial relationships that could be construed as a potential conflict of interest.

## References

[B1] Aiello J. R. Douthitt E. A. (2001). Social facilitation from Triplett to electronic performance monitoring. Group Dyn. 5, 163–180 10.1037/1089-2699.5.3.163

[B2] Aiello J. R. Svec C. M. (1993). Computer montoring of work performance: extending the social facilitation framework to electronic presence. J. Appl. Soc. Psychol. 23, 537–548 10.1111/j.1559-1816.1993.tb01102.x

[B3] Allport F. H. (1920). The influence of the group upon association and thought. J. Exp. Psychol. 3, 159–182 10.1037/h0067891

[B4] Allport F. H. (1924). Social Psychology. New York, NY: Houghton Mifflin Company.

[B5] Amoroso D. M. Walters R. H. (1969). Effects of anxiety and socially mediated anxiety reduction on paired associate learning. J. Pers. Soc. Psychol. 11, 388–396. 10.1037/h0027261 5787027

[B6] Blascovich J. Mendes W. B. Salomon K. (1999). Social “facilitation” as challenge and threat. J. Pers. Soc. Psychol. 77, 68–77. 10.1037/0022-3514.77.1.68 10434409

[B7] Bond C. F. Titus L. J. (1983). Social facilitation: a meta-analysis of 241 studies. Psychol. Bull. 94, 265–292. 10.1037/0033-2909.94.2.265 6356198

[B8] Borden R. J. Hendrick C. Walker J. W. (1976). Affective, physiological, and attitudinal consequences of audience presence. Bull. Psychon. Soc. 7, 33–36 10.3758/BF03337112

[B9] Carver C. S. Scheier M. F. (1981). The self attention-induced feedback loop and social facilitation. J. Exp. Soc. Psychol. 17, 545–568 10.1016/0022-1031(81)90039-1

[B10] Chabaud M. A. Isabel G. Kaiser L. Preat T. (2009). Social facilitation of long-lasting memory retrieval in *Drosophila*. Curr. Biol. 19, 1654–1659. 10.1016/j.cub.2009.08.017 19781943

[B11] Chen S. C. (1937). Social modification of the activity of ants in nest-building. Physiol. Zool. 10, 420–436.

[B12] Cottrell N. B. Wack D. L. Sekerak G. J. Rittle R. (1968). Social facilitation of dominant responses by the presence of an audience and the mere presence of others. J. Pers. Soc. Psychol. 9, 245–250. 10.1037/h0025902 5666972

[B13] Dashiell J. F. (1930). An experimental analysis of some group effects. J. Abnorm. Soc. Psychol. 25, 190–199 10.1037/h0075144

[B14] Elliot E. S. Cohen J. L. (1981). Social facilitation effects via interpersonal distance. J. Soc. Psychol. 114, 237–249 10.1080/00224545.1981.9922753

[B15] Good R. (1973). Social facilitation: effects of performance anticipation, evaluation, and response competition on free association. J. Pers. Soc. Psychol. 28, 270–275. 10.1037/h0035790 4747226

[B16] Guerin B. (1993). Social Facilitation. European Monographs in Social Psychology. Cambridge: Cambridge University Press.

[B17] Harkins S. G. (1987). Social loafing and social facilitation. J. Exp. Soc. Psychol. 23, 1–18 10.1016/0022-1031(87)90022-9

[B18] Harlow H. F. Yudin H. C. (1933). Social behavior of primates. I. Social facilitation of feeding in the monkey and its relation to attitudes of ascendance and submission. J. Comp. Psychol. 16, 171–185 10.1037/h0071690

[B19] Henchy T. Glass D. C. (1968). Evaluation apprehension and the social facilitation of dominant and subordinate responses. J. Pers. Soc. Psychol. 10, 446–454. 10.1037/h0026814 5708047

[B20] Karau S. J. Williams K. D. (1993). Social loafing: a meta-analytic review and theoretical integration. J. Pers. Soc. Psychol. 65, 681–707 10.1037/0022-3514.65.4.681

[B21] Katz D. Schanck R. (1938). Social Psychology. New York, NY: Wiley.

[B22] Kitayama S. Snibbe A. C. Markus H. R. Suzuki T. (2004). Is there any “free” choice? Self and dissonance in two cultures. Psychol. Sci. 15, 527–533. 10.1111/j.0956-7976.2004.00714.x 15270997

[B23] Kraepelin E. (1902). Die Arbeitscurve [The work curve]. Philos. Stud. 19, 459–507.

[B24] Kuraishi S. Kato M. Tsujioka B. (1957). Development of the “Uchida-Kraepelin psychodiagnostic test” in Japan. Psychologia 1, 104–109.

[B25] Markus H. (1978). The effect of mere presence on social: an unobtrusive test facilitation. J. Exp. Soc. Psychol. 14, 389–397 10.1016/0022-1031(78)90034-3

[B26] Markus H. R. Kitayama S. (1991). Culture and the self: implications for cognition, emotion, and motivation. Psychol. Rev. 98, 224–253 10.1037/0033-295X.98.2.224

[B27] McKinney M. E. Gatchel R. J. Paulus P. B. (1983). The effects of audience size on high and low speech-anxious subjects during an actual speaking task. Basic Appl. Soc. Psychol. 4, 73–87 10.1207/s15324834basp0401_6

[B28] Mullen B. Bryant B. Driskell J. E. (1997). Presence of others and arousal: an integration. Group Dyn. 1, 52–64 10.1037/1089-2699.1.1.52

[B29] Nisbett R. E. Peng K. Choi I. Norenzayan A. (2001). Culture and systems of thought: holistic versus analytic cognition. Psychol. Rev. 108, 291–310. 10.1037/0033-295X.108.2.291 11381831

[B30] Ogura Y. Matsushima T. (2011). Social facilitation revisited: increasing in foraging efforts and synchronization of running in domestic chicks. Front. Neurosci. 5:91. 10.3389/fnins.2011.00091 21811436 PMC3142821

[B31] Prokopy R. J. Duan J. J. (1998). Socially facilitated egglaying behavior in Mediterranean fruit flies. Behav. Ecol. Sociobiol. 45, 117–122 10.1007/s002650050419

[B32] Sanbonmatsu D. M. Kardes F. R. (1988). The effects of physiological arousal on information processing and persuasion. J. Consum. Res. 15, 379–385 10.1086/209175

[B33] Schiml P. A. Mendoza S. P. Saltzman W. Lyons D. M. Mason W. A. (1996). Seasonality in squirrel monkeys (*Saimiri sciureus*): social facilitation by females. Physiol. Behav. 60, 1105–1113. 10.1016/0031-9384(96)00134-5 8884940

[B34] Schmitt B. D. Gilovich T. Goore N. Joseph L. (1986). Mere presence and social facilitation: one more time. J. Exp. Soc. Psychol. 22, 242–248 10.1016/0022-1031(86)90027-2

[B35] Simmel E. C. (1962). Social facilitation of exploratory behavior in rats. J. Comp. Physiol. Psychol. 55, 831–833. 10.1037/h0041155 13993046

[B36] Zajonc R. B. (1965). Social facilitation. Science 149, 296–274 10.1126/science.149.3681.269 14300526

[B37] Zajonc R. B. (1980). “Compresence,” in Psychology of Group Influence, ed. Paulus P. B. (Hillsdale, NJ: Erlbaum), 35–60.

[B38] Zentall T. R. Hogan D. E. (1976). Imitation and social facilitation in the pigeon. Anim. Learn. Behav. 4, 427–430 10.3758/BF03214434

[B39] Zentall T. R. Levine J. M. (1972). Observation learning and social facilitation in the rat. Science 178, 1220–1221. 10.1126/science.178.4066.1220 17748985

